# Current status of Crimean‐Congo hemorrhagic fever outbreaks in Uganda and other African countries

**DOI:** 10.1002/hsr2.1383

**Published:** 2023-06-29

**Authors:** Ridwan Olamilekan Adesola, Ahmed Abi Abdi Warsame, Ibrahim Idris

**Affiliations:** ^1^ Department of Veterinary Medicine, Faculty of Veterinary Medicine University of Ibadan Ibadan Nigeria; ^2^ Department of Animal Production and Marketing, Faculty of Agriculture and Environment Gulu University Gulu Uganda; ^3^ Department of Veterinary Medicine, Faculty of Veterinary Medicine Usmanu Danfodiyo University Sokoto Nigeria

**Keywords:** Africa, Crimean Congo Hemorrhagic Fever, outbreak, Uganda

## 1 INTRODUCTION

Following the efforts of Ugandan government to battle the outbreak of Ebolavirus disease in September 2022, there is an outbreak of another zoonotic disease called Crimean‐Congo hemorrhagic fever (CCHF).^1^ CCHF causes widespread epidemics in some regions where tick vector is primarily found. Recent outbreaks in Uganda have been documented, and it is believed that this is due to the quiet spread of the pathogen from animals and ticks to people. The aim of this write‐up is to discuss the current and past outbreaks of CCHF in Uganda and Other African countries and provide necessary recommendations needed to prevent future outbreaks.

## 2 OVERVIEW OF CCHF

CCHF is a viral hemorrhagic fever that belongs to the genus *Orthonairovirus* and family *Nairoviridae*,^2^ with a case fatality rate of 10–40% and mortality rate of 30% after the second weeks of the disease.^1^ This virus is endemic in Africa, Asia, and Europe.^1^ Grassland and shrub in these areas are favorable to ticks which is a key indicator of virus distribution.^3^ The virus's geographic spread is correlated with that of the primary vectors, *Hyalomma* ticks. However, Rhipicephalus and Dermacentor ticks can also carry the CCHF virus, making them a potential source of transmission.^4^ The disease is a zoonotic illness that can infect people by tick bites, or contact with contaminated blood or animal tissues, transmission from human to human is uncommon.^5^ Therefore, individuals working in the livestock industry, animal farms, farmers, and veterinarians are at high risk of infection. Typically, the period of incubation is 1–9 days after a tick bite and 5–13 days after getting into contact with infectious fluids.^2^ Infected individuals manifest a broad range of clinical symptoms which are characterized by fever, headache, vomiting, and diarrhea. Skin rashes, hematoma, epistaxis, and melena might be seen. However, in the advanced stage of the illness, the patient may pass away from shock or organ failure.^6^ Laboratory findings may further reveal some hematological abnormalities. There is no specific treatment for CCHF. The majority of the available CCHF treatment is supportive. The support treatments include; close attention to the fluid and electrolyte balance, ventilation support for appropriate oxygenation, moderate sedation, and hemodynamic support.^7^

## 3 CCHF IN UGANDA AND OTHER AFRICAN NATIONS

In October 2022, Kaberamaido District in Eastern Uganda reported its first case of CCHF, the patient was recently admitted to Kaberamaido General Hospital. The patient is a resident of Abirabira village in the Aperkira sub–country, according to health officials.^8^ Since 2010, Uganda has used a viral hemorrhagic fever surveillance system, where more than 20 sentinel surveillance stations are part of the program and samples from throughout Uganda as well as nearby nations are transported there for examination.^8^ In subsequent years, additional confirmation of outbreaks defined as one or more cases has been made because of the expanded surveillance.^9^ Based on a seroprevalence study conducted in 2020 in Uganda, it was determined that anti–CCHF antibodies are present and very prevalent in cattle.^8^ The infection is linked to a geographical region, growing older, being female, and having a greater tick burden.^10^ In research conducted in 2015, a case of human CCHF was diagnosed, which also confirms the endemicity of the virus in Uganda and demonstrates that tick exposure significantly increases the risk of human infection.^11^ Other African countries, such as South Africa, Egypt, Senegal, Mauritania, Kenya, Sudan, Madagascar, Nigeria, Niger, Ghana, etc., had recorded sporadic outbreaks of CCHF (Table 1). This makes CCHF endemic in Africa, which need surgent interventions.

**Table 1:** CCHF virus epidemic trends in countries across Africa.

Affected countries	Year of outbreaks	Source of Transmission	References
Uganda	2013−2017, 2018 and 2022	Human	[2, 12]
South Africa	1981−2006	Tick, livestock blood and tissue	[13]
Egypt	1981, 2004−2005, and 2012	Human and animal	[14]
Senegal	1970, and 1989	Livestock, and Tick	[15]
Mauritania	1983, 1988, and 2003	Human	[16]
Kenya	1970, and 2000	Tick and human	[17]
Sudan	1988, 1989, and 2008	Nocosomia, and human	[18]
Madagascar	1985, 1988, and 2008−2009	Tick, and Human	[19]
Nigeria	1970, 2010−2014, 2015,	Tick, cattle, goat, and human	[20]
Niger	1984−1988, and 1995	Domestic animals	[21]
Ghana	2011	Tick	[22]

**Figure 1:**
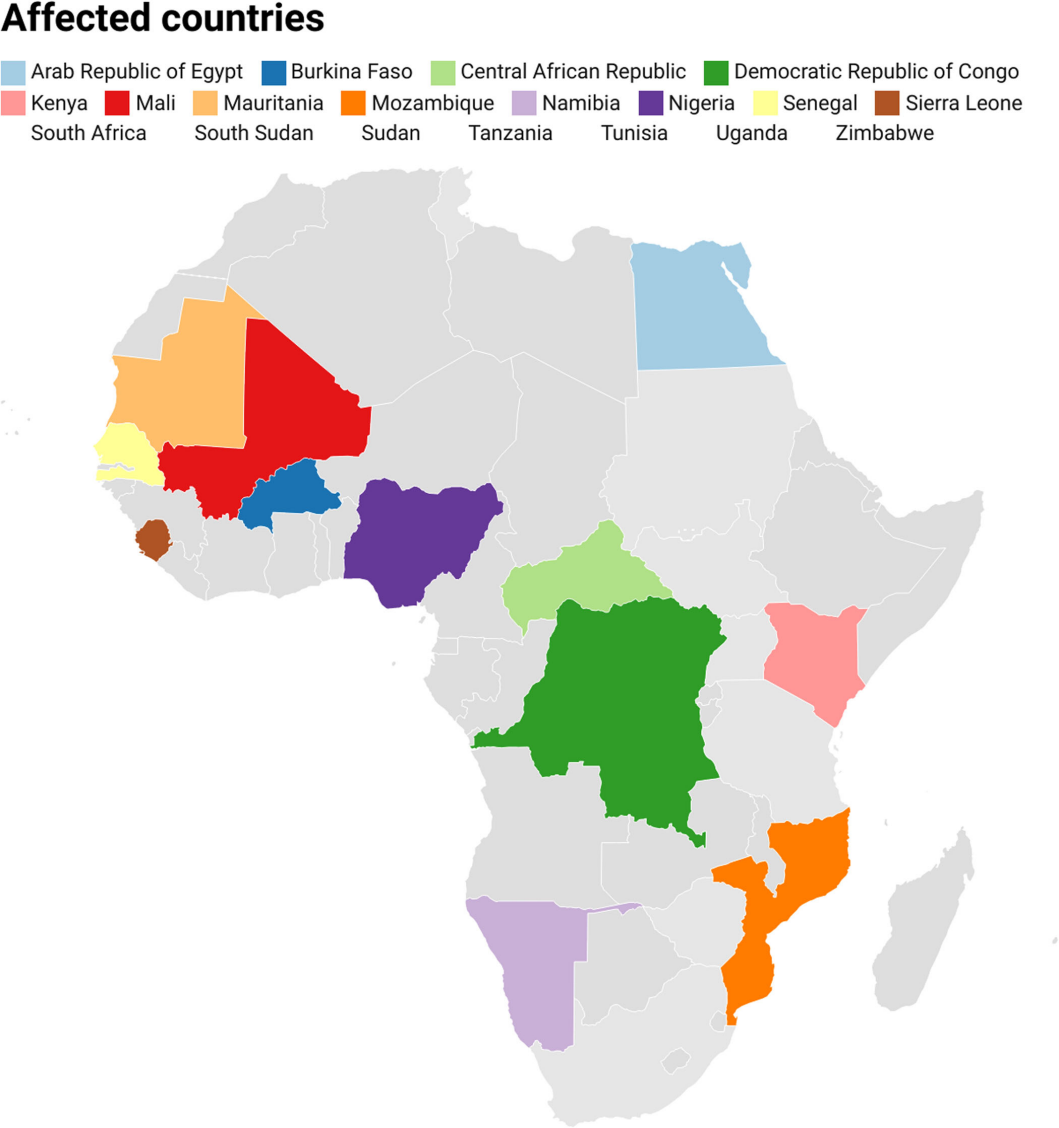
Distribution of Crimean‐Congo Hemorrhagic Fever Cases in Africa from 1956 to 2020.

Animals are affected by CCHF, although they do not exhibit any clinical symptoms. However, 20% of people infected develop clinically fatal conditions.^23^ It is not unexpected that the fatality rate has been estimated to be as high as 60% in certain regions, making it an infection that has to be reported in many nations. CCHF's global economic impact has not been mentioned in any published reports, but other hemorrhagic fevers that are closely related to it are said to have enormous direct and indirect economic costs. CCHF has the potential to cause a pandemic due to its zoonotic nature, however, the disease is endemic in African countries (Figure 1).

## 4 CHALLENGES TO BE ADDRESSED IN UGANDA AND OTHER AFRICAN NATIONS

The public health authorities in Uganda have implemented a tremendous effort in containing the disease spread. However, there is a need for multidisciplinary collaboration in the case of CCHF. The major challenges in the detection of this disease are underreported poor surveillance and misdiagnosis. Some African countries do not engage in surveillance and reporting of the disease except when there is an outbreak, and this is not an effective way of combating infectious diseases, especially of zoonotic importance. Outbreaks may be a result of the international movement of humans or animals via country borders. So also, when there is a single confirmed case of the disease in a certain region, the authorities do not want to report it to international organizations due to political or other reasons. Several infectious diseases of zoonotic origin have the characteristics of viral hemorrhagic fever, so due to the healthcare system in most African countries, these forms of infections such as Ebola, and arboviral infections were misdiagnosed. Another challenge is vaccine availability, some vaccines are available, but vaccine equity and hesitancy are major challenges. Home slaughter is a factor that may increase the risk of a CCHF outbreak, individuals who slaughter animals at home exposed themselves to the risk of being infected when they meet the body fluids of infected animals.

## 5 RECOMMENDATION AND CONCLUSIONS

One of the important tools in fighting this infection is surveillance, despite the timely surveillance system in Uganda, there is a need to put more effort into increasing the surveillance system, not only in Uganda but all over the endemic regions especially African nations, because determining the origin of an outbreak usually made it easy to tackle an outbreak. So also, healthcare workers should be trained on how to handle infected individuals to avoid transmission of the disease in healthcare settings, and they should also be provided with personal protective equipment. Furthermore, Abattoirs and slaughter slabs should be advanced, and the authorities should ensure that infected animals or suspected animals are not slaughtered and should be reported to health authorities immediately. Post‐mortem, ante‐mortem, and meat inspections should be strengthened also. Farmers and animal handlers should be educated on early reports, whenever there is an outbreak in their farms, especially when it is associated with hemorrhagic fever. Environmental health workers and policymakers should sensitize the citizens on hygiene, sanitation, and tick control, controlling ticks in the environment is the best way to combat CCHF. Health authorities, such as the Ministry of Health, Environment, and Agriculture should collaborate in fighting this infection, especially when it comes to the human‐animal interface.

## AUTHOR CONTRIBUTIONS

**Ridwan Olamilekan Adesola**: Conceptualization; data curation; writing—original draft; writing—review and editing. **Ahmed Abi Abdi Warsame**: Data curation; resources; supervision; writing—original draft; writing—review and editing. **Ibrahim Idris**: Conceptualization; data curation; writing—original draft; writing—review and editing.

## CONFLICT OF INTEREST STATEMENT

The authors declare no conflict of interest.

## TRANSPARENCY STATEMENT

The lead author Ridwan Olamilekan Adesola affirms that this manuscript is an honest, accurate, and transparent account of the study being reported; that no important aspects of the study have been omitted; and that any discrepancies from the study as planned (and, if relevant, registered) have been explained.

## Data Availability

Data available on request from the authors.
